# Dr. Vieusseux on Tapping during Pregnancy

**Published:** 1802-01

**Authors:** 

**Affiliations:** Geneva


### To the Editors of the Medical and Phyſical Journal.

Gentlemen,

As you conſider tapping during pregnancy as *a curious fact and worth recording*,* I preſume a ſimilar caſe may not be unacceptable, and might be inſerted in your valuable repoſitory.

In October, 1780, I attended a woman about thirty-five years of age, ill of a bilious fever, which had already laſted ſome days, and appeared complicated with an *aſcites*. Her belly was prodigiouſly ſwelled with evident fluctuation and a great deal of *dyſpnœa*; ſhe voided but very little urine. Thoſe ſymptoms increaſing rapidly from day to day, the fever ſeemed to be almoſt at end, and the dropſy to be the principal diſeaſe. But by the time her *menſes* had ceaſed, a pregnancy was not improbable, though there were no ſigns of it; at leaſt the volume of her belly and the morbid ſtate of her body cauſed by the fever, rendered it impoſſible to judge of it externally.

The difficulty of breathing, with a great deal of anxiety, daily increaſing, the patient now expoſtulated for the operation which ſhe had refuſed when I had propoſed it, ſeeing the inutility of ſeveral diuretics and aperitives I had preſcribed her.

Accordingly, tapping was performed on the right ſide and in the uſual place. After drawing about four pounds of water, it ſtopped of itſelf, and ſomething hard was felt reſiſting againſt the end of the trocar, as if there had been a body or ciſt which the inſtrument could not ſufficiently reach.

We deſiſted for the moment, and the ſurgeon propoſed to make the puncture on the left ſide, and with a longer trocar.

The patient was much relieved by this evacuation, however ſmall; ſhe breathed with leſs difficulty, and voided more urine during ſome days. But all the diſtreſſing ſymptoms returned, and ſhe aſked at any rate to be tapped again; which was done on the left ſide with a larger inſtrument. At firſt we drew about ſixteen pounds of clear yellowiſh water, that began to be mixed with a little blood, which I perceived evidently by receiving it in a cup. We were willing to ſtop, but the woman inſiſted that we ſhould go on, as ſhe felt herſelf relieved: and her pulſe being very good, we let the blood ſtill flow to about the quantity of two pounds, neither the pulſe nor the patient being weakened by the evacuation. We then ſtopped, and the belly did not appear to be much diminiſhed; but

* See London Medical Review and Magazine, vol. iii. page 300.

but the woman was much better. The next day ſhe voided a great quantity of urine; and as the following days it began to diminiſh, the diuretic remedies had more efficacy than they had had before tapping, and the courſe of urine was reſtored. However, the belly was always as ſwelled as before and uniformly hard, and the fluctuation was the ſame.

About fifteen days after the operation, ſhe took ſome purging pills which acted a little briſkly; on the evening ſhe was ſeized with labour pains, and delivered ſucceſſively of three male children, between ten at night and five in the morning. With the firſt child ſhe voided a moderate quantity of water; but with the ſecond and third, ſhe voided a prodigious quantity, and it was only then that the belly ſubſided to its natural volume. She recovered as ſoon and as eaſily as from any other lying-in. The children were judged to be paſt four months old.

This caſe was no doubt a dropſy of the *uterus*, as the belly was reduced to its natural volume only by the delivery, and as the water was evacuated through the *vagina*. The ſucceſs of the operation ſhews not only that tapping may be ſafely practiſed during pregnancy, but that tapping the womb is not dangerous. In the firſt operation the *uterus* was not wounded, and only ſome water externally effuſed was drawn, and probably the body felt at the end of the trocar was the *uterus* itſelf. But in the ſecond operation, made with a longer inſtrument, the uterus was certainly pierced, and the water was drawn from its cavity.

According to the French ſurgeons, the beſt method in theſe caſes is not to tap the belly as in the *aſcites*, but to draw the water by the natural way, viz. through the *vagina* by piercing the membranes and promoting delivery; which ought not to be done but after having previouſly aſcertained the ſtate of pregnancy, by touching the woman when ſtanding, and feeling the ſwelling of the membranes through the dilated *os uteri*. See more on that ſubject in the *Recueil Périodique de la Société de Médicine de Paris*, tom. vi. p. 349; where ſome ſimilar caſes are recorded, moſt of them with two fœtuſes, with many pertinent reflections.

This opinion, however plauſible, might not be ſafeſt in every caſe. In ours there were three fœtuſes, and there was no probability of the woman going to her natural term of delivery. But when there is only one child, may not the tapping of the belly, as in a common *aſcites*, and voiding part of the water contained in the *uterus*, relieve the woman and put her in a ſituation to go to her term, and thus ſave the child, which could not happen if any untimely delivery were promoted by tapping through the *vagina*?

Thus in the caſe related in the Medical Review, tapping caufed no harm, but perhaps contributed to the fafe and timely delivery of the woman.

Even an hæmorrhage is not to be feared, the woman in our cafe loft about two pounds of blood with relief. Some large veffel in the incraffated *parietes* of the *uterus* muft have been opened and have difcharged itfelf through the aperture of the trocar; the wound of the *uterus* was of no confequence.

I remain, &c.

